# Secukinumab-induced Crohn’s disease in a psoriasis patient: a case report highlighting paradoxical reactions to IL-17 inhibition

**DOI:** 10.3389/fimmu.2025.1628461

**Published:** 2025-07-17

**Authors:** Huiling Dai, Chenyi Xu, Yehuang Wang, Kunlan Wu, Yang Zhang, Hao Ge, Yanlan Wu

**Affiliations:** ^1^ Colon and Rectal Surgery, Nanjing Hospital of Chinese Medicine Affiliated to Nanjing University of Chinese Medicine, Nanjing, China; ^2^ Jiangsu Clinical Innovation Center For Anorectal Diseases of Traditional Chinese Medicine (T.C.M), Nanjing Hospital of Chinese Medicine Affiliated to Nanjing University of Chinese Medicine, Nanjing, China; ^3^ School of Clinical Medicine, Jiangsu Health Vocational College, Nanjing, China

**Keywords:** psoriasis, secukinumab, perianal abscess, Crohn’s disease, drug-related adverse reactions

## Abstract

Secukinumab, a monoclonal antibody targeting interleukin-17A, is effective for treating moderate-to-severe plaque psoriasis but may induce paradoxical inflammatory bowel disease, particularly Crohn’s disease. We describe a 41-year-old male with a history of psoriasis who developed new-onset Crohn’s disease accompanied by a perianal abscess after prolonged secukinumab treatment. The patient experienced increased bowel movements and postoperative fever, and colonoscopy revealed diffuse colonic inflammation and deep ulcerations; histopathology confirmed chronic active enteritis with epithelioid granulomas. Infectious causes and other differential diagnoses were excluded. Treatment with corticosteroids and mesalamine led to symptom resolution and normalization of inflammatory markers. This case illustrates a rare but important adverse effect of IL-17A inhibition and emphasizes the dual role of IL-17A in promoting skin inflammation while maintaining intestinal homeostasis. Careful evaluation of gastrointestinal history and close monitoring during therapy are essential to prevent and manage potential intestinal complications.

## Introduction

Secukinumab is a fully human monoclonal antibody that selectively targets interleukin-17A (IL-17A) and is approved for the treatment of adult patients with moderate-to-severe plaque psoriasis who are candidates for systemic therapy or phototherapy (1). By specifically binding to IL-17A, secukinumab inhibits its interaction with receptors, thereby suppressing IL-17A-mediated inflammatory responses and significantly improving psoriatic skin lesions (2).

However, IL-17A also plays a critical role in maintaining intestinal mucosal homeostasis. Its inhibition has been associated with compromised intestinal barrier function, potentially contributing to the onset of inflammatory bowel disease (IBD) (3, 20, 4). In 2020, Fauny et al. (5) conducted a meta-analysis of 38 randomized clinical trials involving 16,690 participants, identifying 12 new-onset IBD cases (5 Crohn’s disease and 7 ulcerative colitis) during a 60-week follow-up period, corresponding to an incidence rate of 2.4‰. Notably, secukinumab was associated with an increasing trend in IBD incidence (6).

This report presents a clinical case of a patient with plaque psoriasis who developed new-onset Crohn’s disease with a perianal abscess during secukinumab therapy. By systematically analyzing the clinical presentation and disease course, this study aims to enhance clinicians’ awareness of this potential adverse event and contribute to improved recognition and management of IL-17 inhibitor-related complications.

## Case report

A 41-year-old male was admitted to our hospital on March 30, 2024, presenting with a one-week history of postoperative abscess accompanied by fever and increased bowel movements. The patient initially experienced perianal discomfort and swelling, followed by rupture of a local mass with purulent discharge and a regular basis in bowel movements 4–5 times per day. He self-administered antibiotics, but the symptoms persisted. He was subsequently evaluated at a local hospital and diagnosed with an anal fistula. One week prior to admission, he underwent surgical treatment at that facility. Postoperatively, he developed a high-grade fever (up to 39.0 °C) and increased bowel frequency (up to 15 times daily). Despite receiving anti-infective treatment and fluid resuscitation, his condition did not improve, prompting transfer to our hospital for further evaluation and management.

The patient had a prior diagnosis of plaque psoriasis in 2021 and had been receiving long-term subcutaneous secukinumab therapy at the outside hospital, with the most recent dose administered on March 6, 2024. He had no personal or family history of inflammatory bowel disease (IBD) but was a known hepatitis B virus (HBV) carrier.

**Figure 1:**
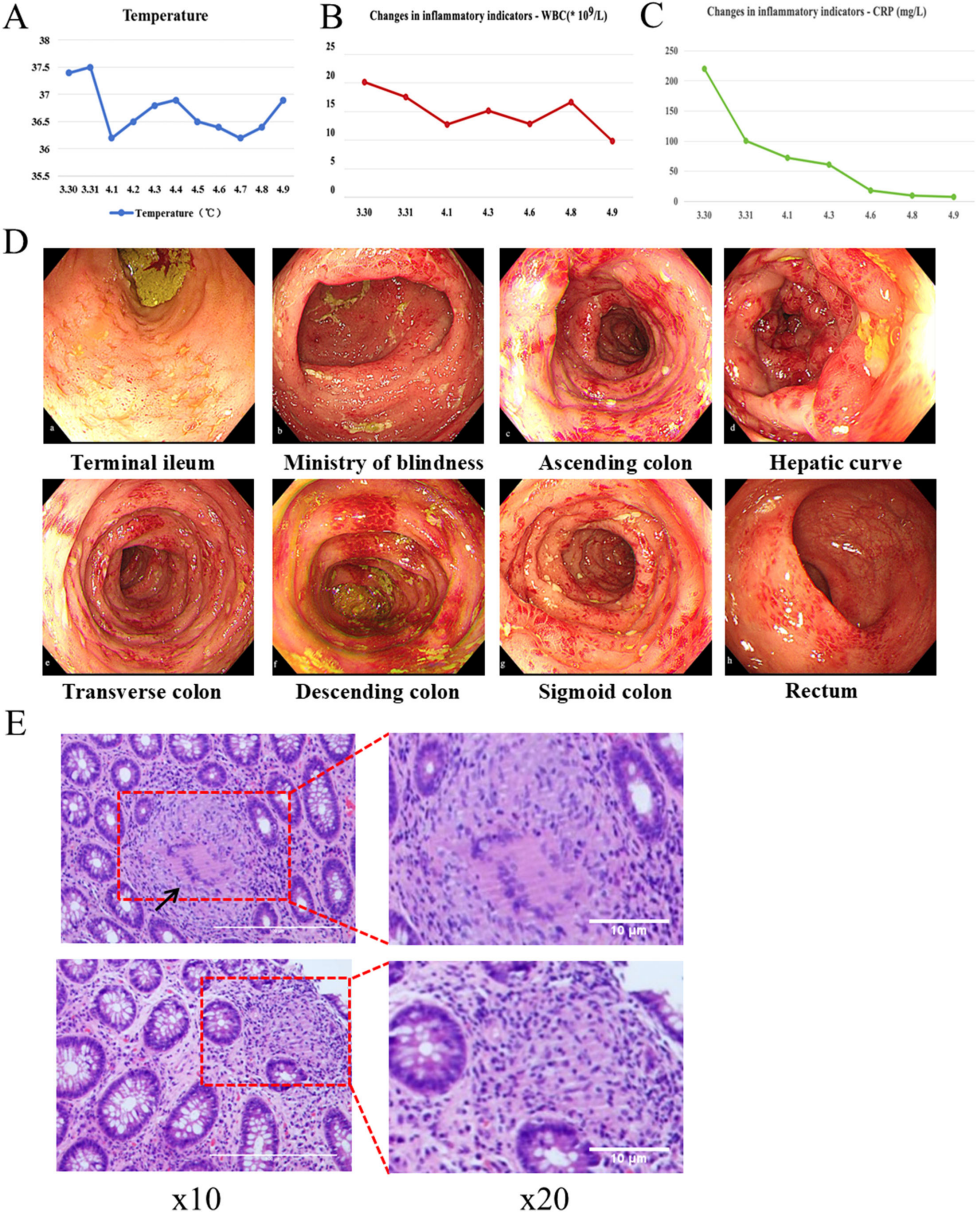
Relevant clinical data during the patient’s hospitalization. **(A)** Changes in body temperature. **(B)** Changes in serum White blood cell (WBC) count. **(C)** Changes in serum C-reactive protein (CRP) concentration. **(D)** Colonoscopy. **(E)** Pathological sections.

Initial laboratory (**Table 1**) workup revealed elevated inflammatory markers, raising suspicion for acute enteritis. The patient was started on compound L-glutamine enteric-coated capsules (two capsules, three times daily), bacillus subtilis and enterococcus faecalis enteric-coated capsules (500 mg, three times daily), cefoperazone-sulbactam (1.5 g, twice daily via intravenous infusion), and montmorillonite powder (3 g, three times daily). By the second day of admission, the patient’s fever had subsided (**Figure 1A**), but bowel movement frequency remained unchanged.

**Table 1:** Pertinent labs throughout patient hospital stay.

Variable Tested	Result
Fecal Calprotectin, ug/g	280.88
Clostridium difficile antigen	Negative
Clostridium difficile antibodies	Negative
PCT, ng/ml	0.08
ESR, mm/60min	38
CMV	Negative
T-SPOT	Negative
HBVDNA, IU/ml	3.29E+04
Stool Culture	Negative

An emergency colonoscopy (**Figure 1D**) performed on April 3 revealed extensive pathology: the terminal ileum showed diffuse mucosal congestion, edema, and scattered erosions. The ileocecal valve was hyperemic but patent, with a visible appendiceal orifice. The cecal base exhibited mucosal congestion, edema, and large, deep ulcers with purulent exudate. The entire colon and rectum demonstrated diffuse congestion, edema, scattered ulcers, vesicular inflammatory changes, and several deep ulcers with purulent coating.

Histopathological analysis (**Figure 1E**) revealed chronic active enteritis with epithelioid granuloma formation, particularly in the left colon. Infectious etiologies were excluded through negative results for Periodic Acid–Schiff (PAS), silver methenamine (PAM), acid-fast staining, and PCR testing for tuberculosis. The findings were consistent with Crohn’s disease. After multidisciplinary consultation, the clinical and pathological features were considered indicative of secukinumab-induced Crohn’s-like disease.

On April 3, the treatment regimen was revised to include bowel rest, parenteral nutrition, intravenous methylprednisolone (40 mg/day), oral mesalamine sustained-release granules (2 g, twice daily), and continued intravenous cefoperazone-sulbactam (1.5 g, twice daily). Given the patient’s HBV carrier status and increased HBV DNA levels, antiviral therapy with oral entecavir (0.5 mg/day) was initiated.

The patient’s symptoms gradually improved. After five days of intravenous corticosteroid therapy, bowel movement frequency decreased to 4–5 times per day. Methylprednisolone was then transitioned to oral administration (40 mg/day; six tablets in the morning and four at noon), and antibiotic therapy was adjusted to oral ornidazole (0.5 g, twice daily). Enteral nutrition support was also introduced.

By the time of discharge on April 9, the patient’s inflammatory markers had significantly improved: C-reactive protein (CRP) decreased to 7.0 mg/L and white blood cell (WBC) count to 9.8 × 10^9^/L (**Figures 1B, C**). After discharge, the patient continued the oral treatment regimen, with a gradual tapering of methylprednisolone per a standardized tapering schedule.

**Figure 2:**
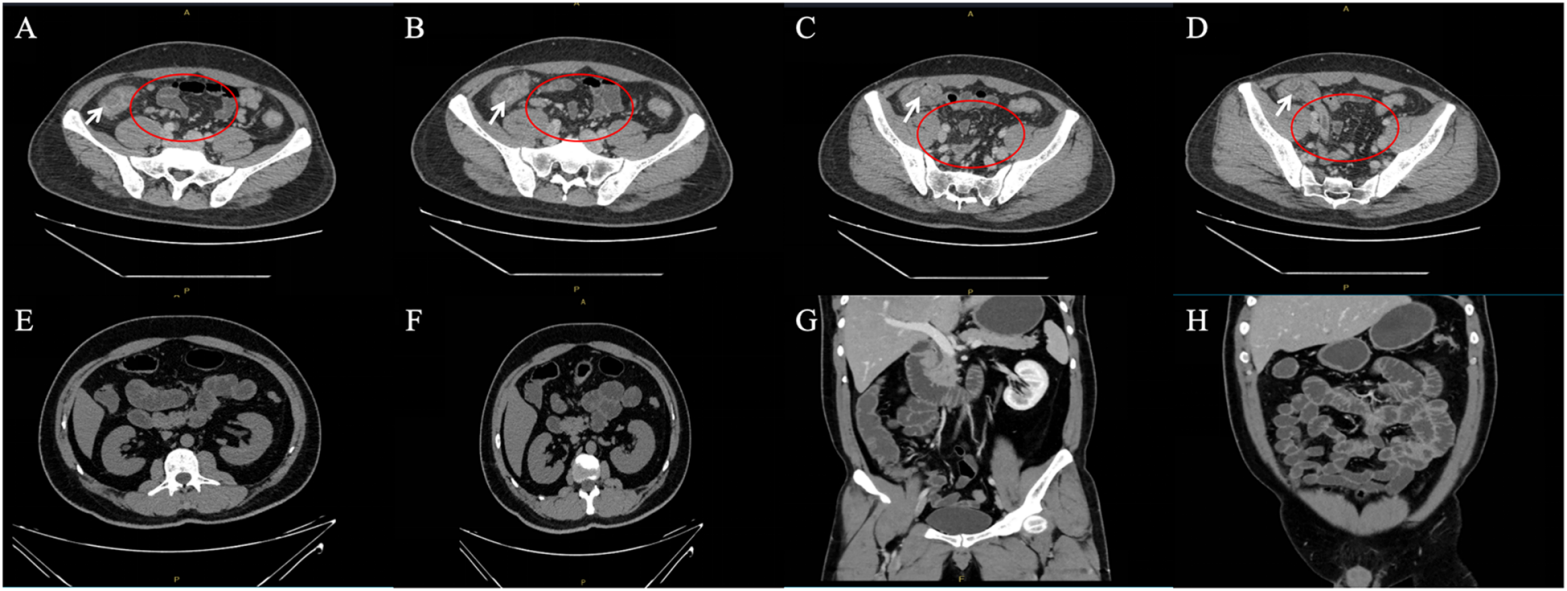
**(A–D)** Enhanced CT scan of the whole abdomen at initial presentation (White arrows show marked edema of the mucosa of the ascending colon; scattered enlarged mesenteric lymph nodes are seen in red circles). **(E–H)** CT scan of the small intestine after 1 year of treatment for Crohn’s disease.

**Figure 3:**
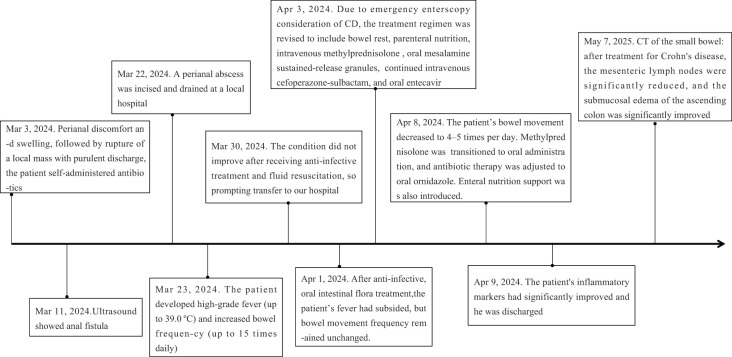
The timeline of the treatment of the patient.

During the follow-up period, the patient reported a recurrence of psoriasis symptoms. Subsequently, a low-dose secukinumab was administered for maintenance treatment, and the symptoms were relieved. After 1 year of treatment for Crohn’s disease, CT of the small intestine showed significant reduction of mesenteric lymph nodes and marked improvement of submucosal edema of the ascending colon compared with Mar 29, 2024 (**Figure 2**). The timeline of the treatment of the patient was presented in **Figure 3**.

## Discussion

Biologic agents have revolutionized the treatment of immune-mediated inflammatory diseases, including psoriasis, by providing targeted immunomodulation. Among them, secukinumab—a monoclonal antibody against IL-17A—has demonstrated significant efficacy in treating moderate-to-severe plaque psoriasis. However, biologics may trigger unique immune-related adverse effects termed paradoxical reactions, where new inflammatory diseases emerge or pre-existing conditions are exacerbated during treatment, often in organs not originally affected (7). In this case, the patient developed new-onset Crohn’s disease after incision and drainage of a perianal abscess during ongoing secukinumab therapy for psoriasis.

Paradoxical IBD associated with IL-17 inhibitors typically manifests weeks to months after treatment initiation, with a reported latency ranging from 1 week to 2 years, and a median onset time around 3 months (8). The patient in this case developed gastrointestinal symptoms approximately 17 days after the last injection, which aligns with the observed timeline in prior reports. Clinical features—including high fever, diarrhea, and perianal inflammation—alongside endoscopic and histopathological findings consistent with Crohn’s disease, support the diagnosis of secukinumab-induced IBD. The prompt improvement following corticosteroid therapy further strengthens the causal link (9, 10).

From a mechanistic perspective, IL-17A plays a dual role in human immunity. While it promotes inflammation in the skin, it also contributes to mucosal barrier integrity in the gastrointestinal tract. IL-17A stimulates epithelial cells to produce tight junction proteins, antimicrobial peptides (such as defensins), and mucins, all of which help maintain barrier function and prevent microbial translocation (11). Blocking IL-17A may thus impair intestinal defense mechanisms, allowing luminal bacteria and antigens to trigger dysregulated immune responses in genetically predisposed individuals, culminating in chronic intestinal inflammation. This tissue-specific function of IL-17A highlights a critical immunological paradox: the same cytokine that drives skin pathology protects against gut inflammation. Interestingly, while IL-17 inhibitors may exacerbate or induce IBD, inhibitors targeting IL-12/23 or IL-23 alone (e.g., ustekinumab, risankizumab) have been approved for both psoriasis and Crohn’s disease, suggesting that upstream modulation of the Th17 axis may be less disruptive to gut homeostasis. This reinforces the need for precision immunotherapy based on disease context and tissue-specific cytokine function.

The observed association between psoriasis and IBD is well documented and supported by shared genetic and immunologic pathways, including polymorphisms in IL23R, NOD2, and CARD9 genes. Genome-wide association studies (GWAS) have identified numerous shared genetic loci between inflammatory bowel disease (IBD) and psoriasis, highlighting key immune-regulatory genes such as IL23R, IL12B, REL, and TYK2 (12). These susceptibility variants converge on inflammatory pathways central to the pathogenesis of both conditions. Furthermore, evidence from twin studies and familial aggregation analyses underscores significant heritability, with high concordance rates observed for both psoriasis and Crohn’s disease (CD) in monozygotic twins (13). These investigations have corroborated GWAS findings, identifying shared susceptibility regions including 6p22, 16q, and 5q33- all functionally linked to immune system regulation (13, 14). In fact, approximately two-thirds of reported secukinumab-induced IBD cases occurred in patients with underlying psoriasis (15). This may reflect both shared pathophysiology and the higher cumulative exposure to IL-17 inhibitors in this population. These observations raise the question of whether screening for IBD risk factors—such as family history, fecal calprotectin levels, or relevant gene variants—should be implemented before initiating IL-17A blockade in psoriasis patients. In individuals with a predisposition to inflammatory bowel disease (IBD), strong blockade of IL-17A by secukinumab may induce immune polarization toward the Th1 axis. This shift is mechanistically significant given that dysregulated Th1 immune activation constitutes a core pathogenic mechanism in Crohn’s disease. The resulting hyperactivation of Th1 pathways serves as a key driver of intestinal inflammation, particularly manifesting as Crohn’s disease-like pathology. Consequently, this provides the mechanistic rationale for exercising caution with IL-17 inhibitors in psoriasis patients at risk of IBD, and explains their contraindication in patients with active IBD.

Based on our follow-up, when the patient was treated with sacituzumab, he had experienced oral Candida infection. This highlights the complex interaction between the intestinal microbiota and IL-17A signaling. Although IL-17 and TH17 cells can cause inflammation and damage the intestinal mucosa, IL-17 and IL-22 also play a protective role in limiting intestinal fungal and bacterial infections. For instance, during Candida infection, IL-17 can induce keratinocytes and immune cells to secrete anti-fungal factors, such as antimicrobial peptides and inflammatory cytokines, enhancing the body’s ability to clear fungi. At the same time, IL-17 can also promote the activation of neutrophils and macrophages, increasing their phagocytic and killing effects on fungi.

Another critical consideration in this case is the patient’s status as a hepatitis B virus (HBV) carrier. There are no strong data regarding the treatment with biologics (especially for the most recent anti-IL-17 and anti-IL-23 drugs) of patients with psoriasis and concomitant viral hepatitis. However, there is a lack of strong evidence regarding the treatment with biologics (especially for the most recent anti-IL-17 and anti-IL-23 drugs) of patients with concomitant viral hepatitis B or C. These more recent biologics treatments appear to have better safety profiles, based on data from both randomized clinical trials and real-life experiences (16, 21, 17). On the other hand, several reports suggest that anti-TNF-α monoclonal antibodies should be avoided in those patients because of the higher risk of serious infections compared with nonbiologic therapies (18). Although there is limited evidence directly linking HBV reactivation to IBD onset, HBV infection has been shown to modulate host immunity, potentially affecting mucosal immune responses. In this patient, the elevation of HBV DNA following secukinumab therapy necessitated the addition of entecavir, consistent with current EASL guidelines recommending prophylactic antiviral therapy in HBsAg-positive individuals undergoing immunosuppression (19). While HBV reactivation could contribute to systemic symptoms, the rapid symptom resolution with corticosteroids, combined with characteristic endoscopic and histopathologic features, strongly supports a primary diagnosis of secukinumab-induced IBD.

This case also highlights the importance of post-marketing surveillance in capturing rare, delayed-onset adverse events not always detected in clinical trials. As biologic therapies become increasingly prevalent, it is imperative that clinicians remain vigilant for paradoxical reactions and report such cases to pharmacovigilance registries. This will contribute to a better understanding of incidence patterns, risk factors, and mechanisms underlying such reactions.

## Conclusion

This case underscores the complex immunological roles of IL-17A and illustrates how its inhibition may lead to severe gastrointestinal consequences in susceptible individuals. A comprehensive evaluation of personal and family history, potential viral co-infections, and subclinical inflammation should be considered prior to initiating IL-17-targeted therapy. Despite the rarity of such cases, they alert clinicians to remain highly vigilant for these adverse reactions and emphasize the importance of timely assessment and diagnosis. Although reports linking secukinumab to new-onset Crohn’s disease remain rare in both domestic and international literature, accumulating clinical evidence—including the present case—suggests a potential association that warrants attention. It is recommended that, before initiating IL-17 inhibitor therapy, patients undergo a thorough pre-treatment screening, including evaluation of gastrointestinal symptoms, family history of IBD, and, where appropriate, fecal calprotectin or occult blood testing. During treatment, the appearance of suspicious gastrointestinal symptoms or fecal calprotectin levels ≥ 250 μg/g should prompt early and systematic investigation to rule out or confirm IBD. Future studies are needed to identify predictive biomarkers and define optimal screening and management strategies to prevent and mitigate paradoxical IBD during IL-17A blockade.

## Limitations

Incomplete Long-Term Follow-Up: The duration of post-treatment follow-up was limited. Longer observation would be helpful to determine whether intestinal inflammation recurs, especially upon tapering or cessation of treatment.

Confounding Factors: Although infectious causes and differential diagnoses were excluded, the possibility of pre-existing subclinical IBD or genetic susceptibility cannot be ruled out. These may have contributed to disease onset independently or synergistically with secukinumab.

## Data Availability

The original contributions presented in the study are included in the article/supplementary material. Further inquiries can be directed to the corresponding authors.
